# Study methodology impacts density-dependent dispersal observations: a systematic review

**DOI:** 10.1186/s40462-024-00478-6

**Published:** 2024-05-21

**Authors:** Nathalie Jreidini, David M. Green

**Affiliations:** 1 Ecomuseum Zoo, Sainte-Anne-de-Bellevue, Montreal, QC Canada; 2 Redpath Museum, McGill University, Montreal, QC Canada

**Keywords:** Conspecific density, Data methodology, Empirical observations, Heterogeneity, Meta-analysis, Migration

## Abstract

The relationship between animal dispersal and conspecific density has been explored in various study systems but results in terms of both the magnitude and the direction of density dependence are inconsistent. We conducted a thorough review of the literature (2000–2023) and found *k* = 97 empirical studies of birds, fishes, herpetofauna (amphibians and reptiles), invertebrates, or mammals that had tested for a correlation between conspecific density and animal dispersal. We extracted categorical variables for taxonomic group, sex, age, migratory behavior, study design, dispersal metric, density metric and variable type, as well as temporal and spatial scale, to test each of their correlation with the effect of density on dispersal (Pearson’s *r*) using linear regressions and multilevel mixed-effect modelling. We found certain biases in the published literature, highlighting that the impact of conspecific density on dispersal is not as widespread as it is thought to be. We also found no predominant trend for density-dependent dispersal across taxonomic groups. Instead, results show that the scale and metrics of empirical observations significantly affected analytical results, and heterogeneity measures were high within taxonomic groups. Therefore, the direction and magnitude of the interaction between density and dispersal in empirical studies could partially be attributed to the data collection method involved. We suggest that the contradictory observations for density-dependent dispersal could be explained by dispersal-dependent density, where density is driven by movement instead, and urge researchers to either test this interaction when applicable or consider this perspective when reporting results.

**Supplementary Information:**

The online version contains supplementary material available at 10.1186/s40462-024-00478-6.

## Introduction

Animal dispersal has long been considered to be impacted by population density [13, 45, 53, 94] especially in the context of colonization and range expansion (Azandémè-Hounmalon et al. 4, Sullivan et al. 2017). Empirical observations for the effect of conspecific density on dispersal, though, have been inconsistent in terms of both the magnitude and the direction of the presumed interaction [61], and difficulty in consistently measuring density and dispersal across study systems may render results unreliable. In theoretical terms, meanwhile, animal dispersal in relation to density has often been modelled mathematically as though a process of diffusion (e.g., [25, 52, 88]) analogous to the diffusion of molecules in gasses and liquids. Yet animals do not move like molecules; they do not disperse by colliding and bouncing off one another, and they can exhibit movements towards areas with higher densities [83, 125]. Therefore, the extent to which animal dispersal and density are correlated, and the nature of this relationship, remain up to question.

It is easy to see that strictly dispersive movements of organisms away from areas of concentration will correlate with a reduction in the density of those organisms. But the direction of causality is not so easily seen, that is, whether initial density is driving the organisms’ movements, or their movements are altering density, or both. Aggregative behaviours performed by animals, such as schooling or herding, increase density, whereas disaggregative behaviors, such as random walk foraging, or spacing behaviours, such as territoriality, decrease density [38]. Numerous species of migrating animals, such as salmon [42] and salamanders [122], will alternate between movement away from a breeding site and aggregation as they return to it, with correlated changes in their density. Alternatively, density may have no perceivable effect on dispersive movements (e.g., [51]), or its effect on dispersal may vary according to a density ‘threshold’ (e.g., [7, 33, 78]) or even appear to be temporary (e.g., [17]). Individual assemblages may therefore be linked to different types of density-dependent dispersal: positive, negative, neutral, and even non-linear [41].

The correlation between density and dispersal may depend on whether there are benefits to living in a group. On one hand, conspecific density may lead to varying levels of intraspecific competition, notably among kin [6, 11, 23, 84], which may in turn favor dispersal and therefore result in positive density-dependence [26, 67, 77, 92, 99, 138]. On the other hand, certain species rely on group living for optimal defense against predators and/or improved foraging efficiency [8, 19, 21, 29, 34, 36, 60, 65], thus leading to a negative correlation between conspecific density and dispersal. In addition, a myriad of behavioral personalities may exist within a group, and individuals may respond to density differently depending on their personality traits. For example, a non-aggressive and shy individual may only approach or settle in a patch that has already been colonized by aggressive and bold conspecifics [30, 39]. Individual characteristics, including behavioral traits, may therefore modulate density-dependent dispersal responses. Nonetheless, empirical studies more commonly focus on the correlation between density and dispersal at the population level.

Dispersal is notoriously difficult to measure in the field as it is often derived from indirect measures that are associated with large uncertainties and potential biases [46]. It can be measured as propensity (the probability that an individual will emigrate), rate (movement distance per unit time), or distance moved. Although these metrics are often used interchangeably in dispersal studies, their relationship with density may differ; high local density might increase individual emigration distances but lead to lower dispersal propensities [79] and, conversely, higher dispersal propensities at higher densities could lead to slower movement rates [2]. Similarly, density can be measured at different life history stages; for example, natal density can be the number of birds in a nest while breeding density the number of nests [14]. Studies have also used proxy measures for density, such as habitat carrying capacity [55], patch area [100], nearest-neighbor distance [51], and even habitat quality [49, 57, 64] on the assumption that higher habitat quality should equate to higher population density [12, 14, 16, 20]. The different density and dispersal metrics therefore could result in incomparable outcomes when it comes to the relationship between density and dispersal.

To assess whether dispersal is density-dependent across study systems and test the impact of methodology on this relationship, we conducted a systematic review of available literature on the effects of conspecific density on dispersal. If density and dispersal are correlated among animals, as would be expected of density-dependent dispersal as a rule, then meta-analytical results should show a convergence among studies toward a strongly supported, weighted mean effect size. However, there is potential for disparities among the results of different studies to be induced by taxonomic and/or methodological differences. Therefore, and unlike existing reviews on density-dependent dispersal [11, 41, 61, 85], we particularly examined the extent to which several categories of study methodology, within and across taxonomic groups, impacted empirical results for the effect of density on dispersal. We explicitly considered heterogeneity and potential reporting of analytical biases, and tested whether reports of density-dependent dispersal were related to taxonomic group, sex, age, migratory behavior, study design, dispersal metric, density metric and variable type, and scales of space and time. We reasoned that if effect size was significantly correlated with one or more of these variables, then the recognition of density-dependent dispersal could be linked to the nature of the study and/or the associated methods employed.

## Methods

### Literature search and data compilation

**Fig. 1:**
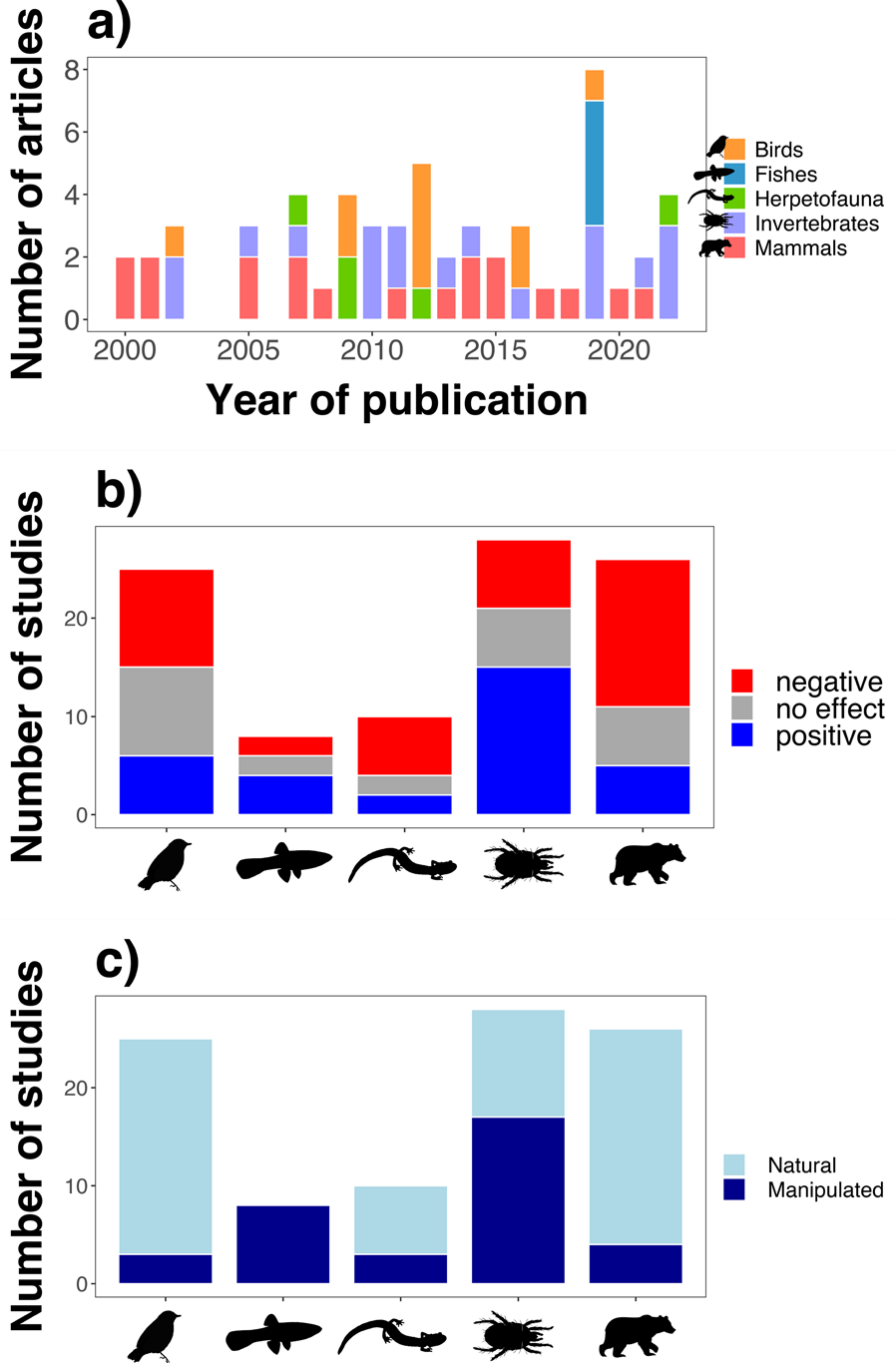
(**a**) Articles included in this review (*n* = 68) categorized by year of publication (2000–2023) and associated studies (*k* = 97) categorized by (**b**) the reported effect of density on dispersal (negative, positive, or no effect) and (**c**) the study design (natural or manipulated), grouped per taxon (Birds, Fishes, Invertebrates, Mammals, and Herpetofauna)

We conducted a thorough review of the literature (peer-reviewed articles only, no preprints) using the Google Scholar database with the keywords “density” and “dispersal” or “emigration” in articles published from January 1st, 2000, through October 1st, 2023, excluding citations (Fig. 1A). We only retained studies that referred to conspecific density rather than heterospecific or interspecific density, measured animal density at the starting point of dispersal rather than at the end point of dispersal, reported a statistical effect specifically of density on dispersal, and reported results from empirical observations and not from simulations or theoretical models. Studies that had prominent confounding variables (such as an effect of body size or patch quality) were also excluded. Finally, certain articles reported results for more than one study. Thus, this search yielded 97 studies in 68 articles that fit our criteria of inclusion (Appendix 1).

### Data extraction and effect size calculation

To derive comparable effect sizes, we extracted the correlation coefficient, Pearson’s *r*, from all studies of density in relation to dispersal where it was available. We used linear correlation results in our analyses, since only a few studies reported a potential non-linear relationship between density and dispersal, and to simplify analyses using a single value of Pearson’s *r* per study. Where not available, we calculated *r* with the information provided [27]. We applied Fisher’s *Z*-transform to linearize *r* values [28], then weighted each value by the reciprocal of its sampling variance [10]. The resulting weighted effect sizes ${Z}_{r}$ were used in subsequent analyses, where a positive value indicates conspecific dispersion (i.e., higher dispersal with increasing density) and a negative value indicates conspecific aggregation (i.e., lower dispersal with increasing density).

### Publication bias

We evaluated publication bias among studies using a funnel plot of weighted effect sizes versus standard error and an Egger’s test for funnel plot asymmetry [32], for all studies individually and within categories. We also applied a trim-and-fill analysis to identify and correct for funnel plot asymmetry [31, 91]. In addition, we assessed publication bias using the Luis Furuyama–Kanamori (LFK) index, as this method is suggested to be more sensitive to potential bias when pooling studies [37]. We did not choose one method over the other as they are both prevalent in the literature and we valued obtaining comparative results. We recalculated the two-tailed probability estimate (*P*) for all studies using sample size (*n*) and Pearson’s *r* to compare statistical significance across studies consistently, with $\alpha$ = 0.05. The ratio of significant to non-significant studies was tested to further identify potential biases resulting from use of differing methodologies.

### Meta-analysis

We defined four categorical variables descriptive of the individual animals involved in the study—*Taxonomic Group*, *Sex*, *Age,* and *Migratory Behavior*—and five categorical variables descriptive of the study methodology—*Study Design*, *Density Metric*, *Dispersal Metric*, *Spatial Scale*, and *Temporal Scale*. We recognized five categories of *Taxonomic Group*: Birds (*k* = 25), Fishes (*k* = 8), Herpetofauna (*k* = 10, consisting of both amphibians and reptiles), Invertebrates (*k* = 28, consisting of insects and arachnids), and Mammals (*k* = 26). The variable, *Sex*, consisted of three levels: males (*k* = 20), females (*k* = 23), and males + females (*k* = 54, studies that reported grouped results for the two sexes), as the ecological determinant of dispersal is often expected to vary between males and females (e.g., in damselflies: [9]). *Age* consisted of two levels: adults (*k* = 63) and juveniles (*k* = 34) and was included as a variable because some animals are thought to disperse more at specific life history stages (e.g., in amphibians: [141], in sparrows: [3]). *Migratory Behavior* described whether the animals were ‘migratory’ (*k* = 40), i.e., if they performed long-distance migratory movements as part of their life history, such as for breeding, mating, or hibernation, or were ‘non-migratory’ (*k* = 57) if they did not perform these movements. This variable was either extracted from the article when reported or assessed through further research on the study species. It was added in this analysis because migratory movements, whether they are performed in groups or not, affect movement tendency and therefore may be confounded with results on dispersal (e.g., migrants disperse farther than residents; [76]).

Among the methodology variables, *Study Design* had two levels: ‘manipulated’ (*k* = 35), including all experimental studies that employed artificial enclosures, microcosms, mesocosms, or laboratory set-ups, and ‘natural’ (*k* = 62) consisting of studies of wild populations in nature. The variable *Dispersal Metric* had three levels: ‘propensity’ (*k* = 36) for studies assessing the probability or frequency of emigration, ‘rate’ (*k* = 20) for studies measuring movement distance per unit time, or ‘distance’ (*k* = 41) for studies recording either average or net distance moved by an animal between two points. Although dispersal is typically defined as any movement that could lead to the consequences of gene flow [84], dispersal is often considered to be composed of three sequential stages—departure, transit, and settlement [5, 11, 24, 62]—which relate to our three *Dispersal Metric* levels, respectively. *Density Metric* also had three levels: ‘natal’ (*k* = 30) for studies measuring density at a birth or developmental site, ‘breeding’ (*k* = 22) for studies measuring density at a breeding site, and ‘population’ (*k* = 45) for studies that assessed abundance of individuals in the whole population. *Density Variable* consequently had two levels: ‘discrete’ (*k* = 71), where density was measured at one time point regardless of spatial and temporal scale, and ‘continuous’ (*k* = 26), where density was measured at multiple time points throughout the temporal period of the study. Studies were also divided based on two levels of *Spatial Scale*: ‘between patches’ (*k* = 48) whereby the start and end point at each patch was recorded, and ‘out of a patch’ (*k* = 49) whereby the starting point of the displacement was recorded in the study, but the settlement point was not. Finally, we categorized *Temporal Scale* of dispersal observations recorded within a year (or less) as ‘intra-annual’ (*k* = 51), between years as ‘inter-annual’ (*k* = 34), and in short-term experimental studies as ‘per trial’ (*k* = 12). Although there are other factors that may have an impact on dispersal, such as sociality and territoriality, we chose to focus on variables relevant to our research question on the effect of methodology on density-dependent dispersal observations.

We tested for significant difference from $\overline{{Z }_{r}}$= 0 across and within taxonomic groups, assuming that each study has its own mean estimate and therefore does not assume homogeneity [70]. Next, to examine the impact of each categorical variable, *Sex*, *Age*, *Migratory Behavior*, *Study design*, *Density Metric*, *Density Variable, Dispersal Metric*, *Temporal Scale*, and *Spatial Scale* on ${Z}_{r}$ we used a meta-analytical approach, a multilevel mixed-effects model with those variables added as fixed effects. The model was fitted via restricted maximum likelihood estimation, with *Taxonomic Group* and *Article* added as random effect variables to account for potential taxa-specific trends and any potential biases for studies extracted from the same article. We used 95% confidence intervals to determine significant differences in effect sizes from zero.

### Heterogeneity testing

We tested for possible sources of heterogeneity, the measure of incompatibility among studies in a meta-analysis. Since a wide variation in density-dependence across studies leads to excessive heterogeneity, we can test what impacts this variation by estimating heterogeneity in different pools of studies [89]. Accordingly, we sorted the studies into pools for analytical purposes, based on all individual and methodology categorical variables, to identify incompatibilities in the results and return lower levels of heterogeneity. Heterogeneity measures *τ* ^2^ (between-study variance or variance of true effects), *I* ^2^ (residual heterogeneity), *H* ^2^ (sampling variability) and *Q* (total residual heterogeneity) were estimated through restricted maximum likelihood. As *I* ^2^ can be compared for studies with different types of outcome data, it was chosen as the preferred measure of heterogeneity [43].* I* ^2^ values were categorized as low (0–30%), moderate (30–75%), and high (75–100%). Thus, if studies are too different to compare within the created groups, then we expect to find high heterogeneity measures, particularly *I* ^2^ values, signifying a difficulty in comparing study outcomes.

All statistical analyses and visualizations were done in R version 4.2.3 [81] and using packages ‘metafor’ [98] and ‘metasens’ [87].

## Results

### Literature review

Our review of the recent literature on density-dependent dispersal indicates that empirical evidence of the existence of density-dependent dispersal is, at best, equivocal. In 40 of the 97 studies we examined, conspecifics attracted each other (i.e., negative density dependence), in 32 other studies, they repelled each other (i.e., positive density dependence), and in the remaining 25 studies, there was no significant density-dependent effect on individual dispersal at all (Fig. 1b). Although more studies report significant results, we found no differences between studies in the proportion of significant versus non-significant results, neither per taxa nor per study category (Appendix 2). In addition, there is no trend between number of articles and the year of publication (Fig. 1a), but there is a clear lack of studies on density-dependent dispersal for Fishes and Herpetofauna relative to other taxonomic groups (Fig. 1b).

Although 26% of studies included in this analysis reported a sex bias, over half of studies, 56%, reported the effect of density on dispersal observed for both male and female individuals together, adding to the difficulty in detecting a potential sex bias. In addition, the effect of density on dispersal was more often explored in females than males due to the relevance of dispersing genes (24% of studies on female dispersal, 21% on male dispersal), especially in invertebrates (e.g., [107, 116]). The direction of the effect of density on dispersal (e.g., fruit flies show female-biased density-dependence at low densities and male-biased at high densities, [66]) or linearity (e.g., linear effect in female leopards and non-linear, quadrative effect in males, [33]) also differed between sexes, although not enough studies observed or reported this difference to explore it further. As for age differences in dispersal results, 10% of studies reported an age-bias but only 35% of studies tested the effect of density on dispersal in juveniles alone.

There was no mention of migratory behavior in most articles, which may have led to a bias in the definition of different movement types, including dispersal, in the associated studies. We therefore added the categorical variable for *Migratory Behavior* based on further research on each species in question. We did not find a significant correlation between *Migratory Behavior* and *Taxonomic Group* (${\chi }^{2}$ = 7.357, *df* = 4, *P* = 0.118), but instead found that across groups over half of species studied, 59%, do not perform migratory movements during their life history, while the opposite was true for Birds (60% are migratory).

As for extracted categorical variables related to study methodology, there was a bias in the measurement of both density and dispersal within taxonomic groups. There was a significant correlation between *Study Design* and *Taxonomic Group* (${\chi }^{2}$ = 32.814, *df* = 4, *P* < 0.001), where studies were more likely to use a manipulated setup in Fishes and Invertebrates, probably due to the difficulties associated with finding and tracking species in these groups, and a natural setup was more commonly used in Birds, Herpetofauna, and Mammals (Fig. 1c). Consequently, *Study Design* significantly impacted effect size ${Z}_{r}$ across groups (${\chi }^{2}$ = 12.194, *df* = 2, *P* = 0.002).

**Fig. 2:**
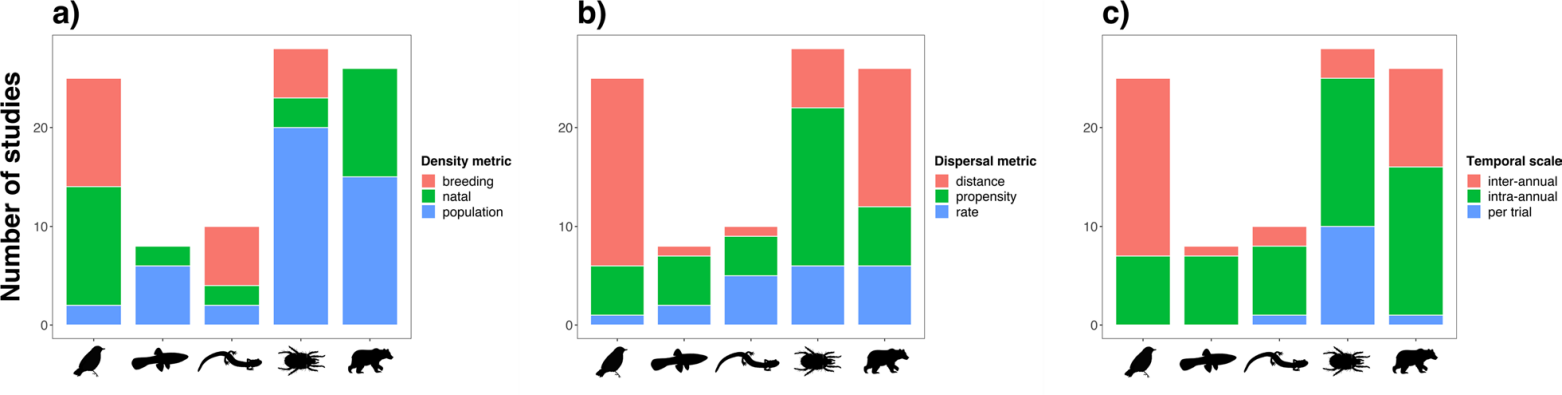
Studies (*k* = 97) grouped per taxon (Birds, Fishes, Invertebrates, Mammals, and Herpetofauna) and categorized by (**a**) density metric (breeding, natal, population), (**b**) dispersal metric (distance, propensity, rate), and (**c**) temporal scale (inter-annual, intra-annual, per trial)

Studies significantly differed in their *Density Metric* (${\chi }^{2}$ = 42.125, *df* = 8, *P* < 0.001), *Dispersal Metric* (${\chi }^{2}$ = 30.058, *df* = 8, *P* < 0.001) and *Temporal Scale* of observations (${\chi }^{2}$ = 39.790, *df* = 8, *P* < 0.001) based on *Taxonomic Group* (Fig. 2). This was not the case for the remaining categorical variables for study methodology *Density Variable* (${\chi }^{2}$ = 4.476, *df* = 4, *P* = 0.345) and *Spatial Scale* (${\chi }^{2}$ = 7.356, *df* = 4, *P* = 0.118) (Appendix 2). However, only *Density Metric* significantly impacted effect size ${Z}_{r}$ across groups (${\chi }^{2}$ = 12.381, *df* = 4, *P* = 0.015), where studies measuring breeding density reported generally stronger and negative density-dependence effects while studies measuring natal or population density reported slightly positive density-dependence effects when averaging effect size, $\overline{{Z }_{r}}$, across groups (Fig. 4).

### Publication bias

Funnel plot analysis provided evidence for slight publication bias due to an underreporting of negative density-dependence (Suppl. Figure S1). Trim-and-fill estimation indicated that deviation from symmetry was slightly skewed toward lower, negative effect sizes and returned a corrected mean value of $\overline{{Z }_{r}}$= − 0.149 compared to the actual value $\overline{{Z }_{r}}$ of − 0.022 $\pm$ 0.047 (Appendix 2). The distribution of weighted effect sizes ${Z}_{r}$ did not significantly deviate from symmetry when all 97 studies were considered (Egger’s test: *t* = 1.76, *P* = 0.08), although some study variables were found to contribute some asymmetry (Tables 1, 2). Furthermore, the high sensitivity of the LFK method detected asymmetry within more categories than with Egger’s test (Table 2), where an across-studies LFK index of 1.05 suggests minor yet significant asymmetry across categorical variables.

**Table 1:** Results for multilevel mixed-effects meta-analysis for effect size ${Z}_{r}$ with *Taxonomic Group* and *Article* as random effects, and all remaining study categories as fixed effects

Study category	Multilevel mixed-effect model		
	Estimate ± SE	z-value	*P*
**Intercept**	– **0.613 ± 0.180**	**-3.408**	< 0.001***
Sex			
Males + Females	**–**	**–**	**–**
Males	– 0.071 ± 0.092	– 0.765	0.444
Females	– 0.141 ± 0.094	– 1.499	0.134
Age			
Adults	**–**	**–**	**–**
Juveniles	0.034 ± 0.090	0.383	0.701
Migratory behavior			
Migratory	**–**	**–**	**–**
** Non-migratory**	**0.225 ± 0.103**	**2.159**	0.031*
Study design			
Natural	**–**	**–**	**–**
Manipulated	0.174 ± 0.125	1.384	0.166
Density metric			
Breeding	**–**	**–**	**–**
** Natal**	**0.321 ± 0.146**	**2.205**	0.028*
** Population**	**0.323 ± 0.141**	**2.280**	0.022*
Density variable			
Continuous	**–**	**–**	**–**
Discrete	0.146 ± 0.120	1.220	0.223
Dispersal metric			
Propensity	**–**	**–**	**–**
**Distance**	**0.200 ± 0.103**	**1.932**	0.050*
Rate	0.071 ± 0.125	0.565	0.572
Temporal scale			
Intra-annual	**–**	**–**	**–**
** Inter-annual**	**0.340 ± 0.116**	**2.934**	0.003**
Per trial	– 0.040 ± 0.161	– 0.246	0.805
Spatial scale			
Between patches	**–**	**–**	**–**
Out of patch	– 0.033 ± 0.101	– 0.326	0.745

^*^ *P* significant difference at $\alpha$ = 0.05; ** *P* significant difference at $\alpha$ = 0.01; *** *P* significant difference at $\alpha$ = 0.001

**Table 2:** Results from Egger’s test and LFK test for publication bias (asymmetry) for all study categories

Study category	Egger's test		LFK test
	*t*	*P*	*index*
Taxonomic group			
Birds	– 0.32	0.752	– 0.26
** Fishes**	**3.00**	0.024*	8.6**
Herpetofauna	– 0.96	0.367	– 1.15*
Invertebrates	0.25	0.807	0.72
Mammals	0.1	0.923	– 0.58
Sex			
Males	– 0.22	0.829	0.31
** Females**	1.26	0.221	1.72*
Males + Females	0.40	0.688	0.80
Age			
** Adults**	1.64	0.107	1.41*
** Juveniles**	– 2.14	0.040	– 1.82*
Migratory behavior			
Migratory	– 1.07	0.290	– 0.84
** Non-migratory**	**2.54**	0.014*	2.49**
Study design			
Natural	– 0.81	0.418	– 0.88
** Manipulated**	**3.84**	< 0.001***	6.24**
Density metric			
** Breeding**	– 1.64	0.116	– 1.39*
** Natal**	– 0.92	0.365	– 1.06*
** Population**	**2.54**	0.015*	2.13**
Density variable			
Discrete	– 1.03	0.309	– 0.20
Continuous	1.56	0.133	– 0.12
Dispersal metric			
Distance	0.75	0.460	0.63
** Propensity**	**2.03**	0.05*	2.88**
** Rate**	– 2.00	0.061	– 1.44*
Temporal scale			
Inter-annual	0.17	0.870	0.33
Intra-annual	0.97	0.339	0.73
** Per trial**	**3.03**	0.013*	5.62**
Spatial scale			
** Between patches**	1.59	0.118	1.49*
Out of patch	– 0.25	0.805	– 0.37

Egger’s test: * *P* significant at $\alpha$ = 0.05; ** *P* significant at $\alpha$ = 0.01; *** *P* significant difference at $\alpha$ = 0.001

LFK test index: * minor asymmetry (|index|> 1); ** major asymmetry (|index|> 2)

### Effect sizes and meta-analysis

**Fig. 3:**
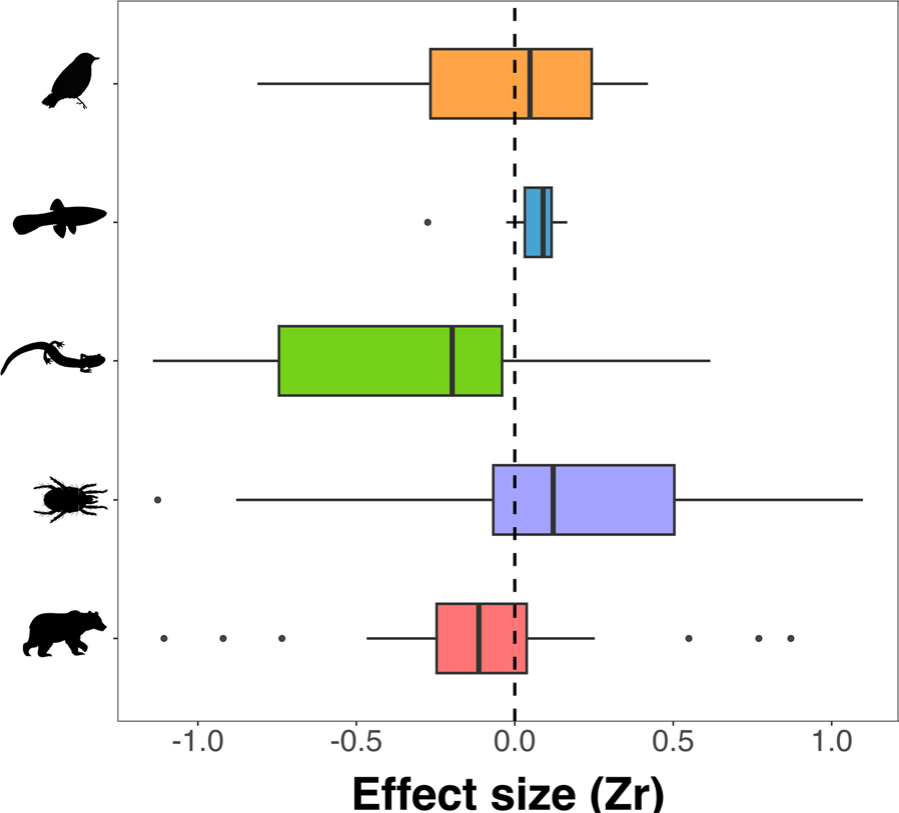
Forest plot for density-dependent dispersal effect sizes (${Z}_{r}$) per taxonomic group

Variation in the mean effect size $\overline{{Z }_{r}}$ of density on dispersal varied largely in both sign and magnitude within and between *Taxonomic Groups* (Fig. 3; Appendix 2). The mean effect size was positive in Birds, Fishes, and Invertebrates, while negative in Herpetofauna and Mammals. However, this interaction was not significant within any taxonomic group, indicating a difficulty in obtaining a clear density-dependence signal, if any, without taking into consideration other variables.

**Fig. 4:**
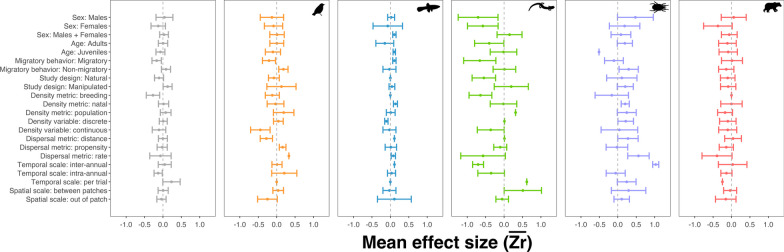
Forest plots for density-dependent dispersal mean effect sizes ($\overline{{Z }_{r}}$) for individual and methodology categorical variables, per taxonomic group (left to right: All groups, Birds, Fishes, Herpetofauna, Invertebrates, Mammals). 95% Confidence Intervals are plotted

Results from the multilevel mixed-effect model further showed that *Migratory Behavior*, *Density Metric*, *Dispersal Metric*, and *Temporal Scale* were all significant predictors of ${Z}_{r}$, with *Taxonomic Group* and *Article* considered as random effect variables (Table 1). Migratory animals were more likely to exhibit negative density-dependence and vice versa for non-migratory animals, particularly in Birds, Herpetofauna, and Invertebrates (Fig. 4). *Sex* and *Age* were not significant predictors of ${Z}_{r}$, neither across nor within groups, but breeding and natal densities had opposite effects on dispersal—breeding density was negatively correlated with dispersal in Birds, Herpetofauna, and Invertebrates, while natal density was positively correlated with dispersal in Birds, Fishes, Herpetofauna, and Invertebrates (Fig. 4). Nonetheless, $\overline{{Z }_{r}}$ values were not significantly different from zero (Appendix 2).

### Heterogeneity testing

Heterogeneity estimates obtained for variables across all *Taxonomic Groups* at once were moderate (*I* ^2^ ≈ 65%, *H* ^2^ ≈ 3) yet significant (*P-*value for *Q* < 0.05) (Appendix 2). Heterogeneity varied between *Taxonomic Groups*, but was generally low (*I* ^2^ < 50%, *H* ^2^ < 2.0) and not significant (*P-*value for *Q* > 0.05) across categorical variables within groups, with low variance of true effects (τ ^2^ < 0.1) (Appendix 2). The only exception was for Invertebrates, where the significantly high heterogeneity across variables (*I* ^2^ > 78%, *H* ^2^ > 2.5, *P-*value for *Q* < 0.05, τ ^2^ > 0.1) was probably due to the widely different families included in this taxonomic group for this study.

## Discussion

Our systematic review and associated analyses show that there is no empirical consensus on whether conspecific density is correlated with dispersal, neither positively nor negatively, emphasized by the high heterogeneity obtained across taxonomic groups. As for results within taxonomic groups, effect sizes varied greatly in magnitude and direction, but heterogeneity estimates were lower than across groups. The metrics chosen to measure density and dispersal, along with the temporal scale of observations were especially found to be significant predictors of the effect of density on dispersal. Our findings are therefore consistent with the hypothesis that the perceived interaction between conspecific density and dispersal can be, at least partially, the result of study methodology.

The process of animal dispersal spans a wide range of spatial and temporal scales [72] and density patterns are spatially variable [68]. However, temporal and spatial scale of displacements are generally acknowledged as the main difficulties in obtaining a unified concept in movement ecology [44]. The settlement point following emigration may often be unknown due to the relatively large scale of the movement path [71]. Logically, animals should be able to disperse farther if allowed more time to do so, but movement paths can be more intricate, with a certain degree of directional variation, at large time scales [80]. We found that measurements of dispersive movements between years were generally reported as positively density dependent as opposed to movements within a year. We also found that significant and positive effects of density on dispersal were more likely for studies with manipulated population parameters. Hence, there may simply be a higher probability for dispersal to occur with increasing time elapsed between encounters, and/or under non-natural density conditions.

Our results also highlight that empirical observations may result from density being movement-dependent rather than movement being density-dependent. Similar to how social organizations can shift throughout an organism’s life history, we found that breeding density, typically requiring aggregation, decreased dispersive tendency, whereas natal density, often followed by disaggregation, increased dispersive tendency. This corroborates with the physical principle of phase separation, or movement-driven self-organization, where the net movement of a species switches between aggregation and disaggregation as a function of its own local density (Cahn and Hillard 18). Aggregation by individual movement is a widely described phenomenon [97] and some species move in groups according to a specific ‘leading point’ [74]. In addition, as the costs associated to dispersal could affect its relationship with density [95], density dependence may only truly be tested in populations where density fluctuates and meets a specific threshold. Nonetheless, too few studies included in our study measured density over a continuous scale, and even fewer reported a density threshold where effects go from negative to positive or vice versa (e.g., [7, 33]) to explore the potential shift in the correlation between dispersal and density over space and/or time. More studies should measure the opposite interaction, the effect of dispersal on density (e.g., Jeong and Kim [1, 47]), to better understand how the two are correlated in different systems.

Migration is a different process from dispersal and we only included studies on dispersive movements in this analysis, but species that typically migrate as part of their life history could move differently in response to conspecific density levels. Migratory tendency can vary within taxonomic group and even within species, but migratory movements generally occur as a response to seasonality and/or for breeding [22, 73]. Migratory species of birds and insects generally undertake relatively large seasonal movements, in groups, often as the entire population [40, 86]. Our results comply with this, as migratory species in those taxa were found to exhibit negative density dependence, while non-migratory species exhibited positive density dependence. However, the opposite was true for fishes and mammals, where migratory animals exhibited positive density dependence. As fishes generally migrate for maturation (e.g., *Salmo salar,* [93], and *Argyrosomus japonicus*, [92]), and many mammals migrate individually for hibernation [59], these animals may be conditioned to seek lower densities.

Comparable to a recent review conducted on density-dependent dispersal in small mammals [85], we find that the reported observations on the effect of density on dispersal are limited and do not allow for the comparison or generalization of dispersal behavior across systems, even within taxonomic group. Another recent review reported that during 2009–2018, most studies on animal movement were in relation to external factors, but of those, only 38% were on movement in relation with other animals, conspecific or heterospecific [50]. Thus, the actual proportion of studies testing the impact of conspecific density on dispersal is relatively low, although we found enough studies between 2000–2023 to test the potential impact of study methodology on density-dependent dispersal observations. However, the relatively low number of empirical evidence for density-dependence leads theoretical studies to either assume different density-dependence scenarios as dispersal strategies (e.g., [69]) or, as seen in many founding theoretical studies on dispersal, assume density-independence [54, 75].

Studies often refer to density to explain other findings related to movement without having actual density measurements to test the direct correlation between density and dispersive movements (e.g., [15, 48, 82]). Similarly, certain studies also use dispersal to justify their results for density or abundance, again without obtaining measurements for dispersive movements (e.g., [63]). Therefore, although density-dependent dispersal is considered a widespread strategy, many studies could not be included in this systematic review as their conclusions are not based on statistical results for the impact of density on dispersal.

## Conclusion

In this systematic review of literature testing the effect of density on dispersal (2000–2023), we show that empirical observations for density-dependent dispersal may be impacted by more than just the characteristics of the population and system under study. We suggest that the contradictory observations for density-dependent dispersal could be explained by dispersal-dependent density in addition to study methodology. As movement occurs as part of the animals’ daily lives regardless, movements within or between patches could impact population density measures. Empirical studies should make sure to place their results within the context of their study system and consider the two-way interaction between movement and density when discussing findings.

### Supplementary Information

Additional file 1.

## Data Availability

The dataset used in this study is available on the Dryad Data Repository (https://doi.org/10.5061/dryad.cz8w9gj6c).

## Appendix 1: Study species and data sources used in systematic review.

**Table:**

Taxon	Study species		*K*	Article reference(s)
	Scientific name	Common name		
Amphibians	*Ambystoma annulatum*	Ringed Salamander	1	Ousterhout and Semlitsch 138
Amphibians	*Anaxyrus fowleri*	Fowler’s Toad	1	Jreidini and Green 51
Amphibians	*Triturus cristatus*	Northern Crested Newt	2	Cayuela et al. 21
Birds	*Athene cunicularia*	Burrowing Owl	1	Luna et al. 130
Birds	*Ciconia ciconia*	White Stork	1	Itonaga et al. 124
Birds	*Hirundo rustica*	Barn Swallow	2	Scandolara et al. 145
Birds	*Junco hyemalis*	Dark-eyed Junco	1	Liebgold et al. 128
Birds	*Lagopus lagopus*	Willow Ptarmigan	2	Brøseth et al. 14
Birds	*Milvus migrans*	Black Kite	2	Forero et al. 34
Birds	*Neophron percnopterus*	Egyptian Vulture	3	Serrano et al. 144
Birds	*Parus major*	Great Tit	1	Nicolaus et al. 135
Birds	*Passer domesticus*	House Sparrow	1	Pärn et al. 77
Birds	*Petroica traversi*	Black Robin	2	Paris et al. 139
Birds	*Pica pica*	Eurasian Magpie	2	Molina-Morales et al. 67
Birds	*Picoides borealis*	Red-cockaded Woodpecker	1	Pasinelli and Walters 140
Birds	*Setophaga ruticilla*	American Redstart	2	McKellar et al. 65
Birds	*Somateria mollissima*	Common Eider	1	Öst et al. 137
Birds	*Sturnus unicolor*	Spotless Startling	1	Fuentes et al. 36
Birds	*Sula nebouxii*	Blue-footed Booby	2	Kim et al. 125
Fishes	*Argyrosomus japonicus*	Mulloway	1	Taylor et al. 92
Fishes	*Oncorhynchus tshawytscha*	Chinook Salmon	1	Connor et al. 26
Fishes	*Poecilia reticulata*	Guppy	4	De Bona et al. 113
Fishes	*Salmo salar*	Atlantic Salmon	2	Einum et al. 117, Teichert et al. 93
Mammals	*Capreolus capreolus*	Roe Deer	1	Gaillard et al. 120
Mammals	*Castor fiber*	Eurasian Beaver	1	Mayer et al. 133
Mammals	*Cervus elaphus*	Red Deer	1	Loe et al. 129
Mammals	*Equus ferus caballus*	Horse	1	Marjamäki et al. 132
Mammals	*Giraffa camelopardalis tippelskirchi*	Masai Giraffe	1	Bond et al. 108
Mammals	*Lepus europaeus*	European Hare	2	Avril et al. 104, Bray et al. 12
Mammals	*Lynx lynx*	Eurasian Lynx	1	Zimmermann et al. 147
Mammals	*Martes pennanti*	Fisher	1	Carr et al. 20
Mammals	*Microtus oeconomus*	Tundra Vole	3	Aars and Ims 101 , Andreassen and Ims 103 , Ims and Andreassen 123
Mammals	*Mustela furo*	Ferret	1	Caley and Morriss 110
Mammals	*Odocoileus virginianus*	White-tailed Deer	1	Lutz et al. 58
Mammals	*Panthera pardus*	Leopard	2	Fattebert et al. 33
Mammals	*Peromyscus boylii*	Brush Mouse	2	Mabry 60
Mammals	*Peromyscus maniculatus*	Deer Mouse	1	Denomme-Brown et al. 29
Mammals	*Suricata suricatta*	Meerkat	1	Maag et al. 131
Mammals	*Ursus americanus*	American Black Bear	3	Kopsala et al. 126
Mammals	*Ursus arctos*	Brown Bear	2	Støen et al. 146
Reptiles	*Anolis sagrei*	Brown Anole	2	Calsbeek 19
Reptiles	*Lacerta vivipara*	Viviparous Lizard	1	Cote and Clobert 112
Reptiles	*Podarcis sicula*	Italian Wall Lizard	1	Vignoli et al. 99
Invertebrates	*Anoplophora glabripennis*	Asian Long-horned Beetle	1	Bancroft and Smith 105
Invertebrates	*Bembidion atrocaeruleum*	Ground Beetle	1	Bates et al. 106
Invertebrates	*Calopteryx splendens*	Banded Demoiselle	1	Chaput-Bardy et al. 111
Invertebrates	*Carpetania matritensis*	Earthworm sp.	1	Navarro et al. (2022)
Invertebrates	*Coenagrion mercuriale*	Southern Damselfly	1	Rouquette and Thompson 143
Invertebrates	*Corbicula fluminea*	Asian Clam	1	Pernecker et al. 78
Invertebrates	*Drosophila melanogaster*	Common Fruit Fly	1	Betini et al. 8
Invertebrates	*Erigone atra*	Dwarf Spider	3	De Meester and Bonte 114
Invertebrates	*Maculinea teleius*	Scarce Large Blue	2	Nowicki and Vrabec 136
Invertebrates	*Melitaea cinxia*	Glanville Fritillary	3	Enfjäll and Leimar 119, DiLeo et al. 116
Invertebrates	*Metrioptera brachyptera*	Bog Bush-cricket	2	Brunzel 109
Invertebrates	*Notonecta undulata*	Grousewinged Backswimmer	1	Baines et al. 7
Invertebrates	*Pacifastacus leniusculus*	Signal Crayfish	1	Galib et al. 121
Invertebrates	*Pardosa purbeckensis*	Saltmarsh Wolf Spider	2	Puzin et al. 142
Invertebrates	*Parnassius mnemosyne*	Clouded Apollo	1	Kuussaari et al. 127
Invertebrates	*Paroxyna plantaginis*	Fruit Fly	2	Albrectsen and Nachman 102
Invertebrates	*Tetranychus sp.*	Spider Mite	3	Azandeme-Hounmalon et al. 4, Bitume et al. 107, De Roissart et al. 115
Invertebrates	*Tribolium castaneum*	Red Flour Beetle	1	Endriss et al. (2019)

## Appendix 2: Summary of results across and within all taxonomic groups (All groups, Birds, Fishes, Herpetofauna, Invertebrates, Mammals) for equality of proportions analysis, effect sizes, and heterogeneity of weighted effect sizes (${Z}_{r}$) obtained from random effect meta-analysis.

**Table:**

Study category	No. studies (*k*)	Total sample size (*n*)	No. probability estimates		Equality of proportions		Effect size		Heterogeneity			
			Sig.	Not Sig.	*X*^2^	*P*	$\overline{r }$	$\overline{{Z }_{r}}$*±* SE	τ^2^	*I*^2^ (%)	*H*^2^	*Q*
All groups												
	97	68390	72	25			– 0.017	– 0.022 ± 0.047	0.12	68.72	3.20	276^a^
Taxonomic Group:					1.920	0.75			0.11	65.16	2.87	247^a^
Birds	25	8359	16	9			– 0.039	– 0.051 ± 0.071				
Fishes	8	31150	6	2			0.043	0.042 ± 0.050				
Herpetofauna	10	2618	8	2			– 0.260	– 0.326 ± 0.170				
Invertebrates	28	10815	22	6			0.141	0.166 ± 0.104				
Mammals	26	15448	20	6			– 0.089	– 0.101 ± 0.086				
Sex:					2.560	0.28			0.12	68.24	3.15	267^a^
Males	20	14814	14	6			– 0.030	0.041 ± 0.117				
Females	23	25264	20	3			– 0.114	– 0.128 ± 0.100				
Males + Females	54	28312	38	16			0.030	0.029 ± 0.060				
Age:					3.260	0.07			0.12	69.06	3.23	275^a^
Adults	63	59124	37	14			0.004	0.002 ± 0.065				
Juveniles	34	9266	15	15			– 0.055	– 0.068 ± 0.062				
Migratory Behavior:					0.008	0.93			0.11	66.23	2.96	262^a^
Migratory	40	22833	29	11			– 0.143	– 0.171 ± 0.065				
Non-migratory	57	45557	43	14			0.072	0.082 ± 0.063				
Study Design:					0.063	0.801			0.12	68.22	3.15	274^a^
Natural	62	30113	45	17			– 0.091	– 0.106 ± 0.063				
Manipulated	35	38277	27	8			0.115	0.126 ± 0.062				
Density Metric:					2.609	0.271			0.10	63.72	2.76	247^a^
Breeding	22	7859	19	3			– 0.226	– 0.271 ± 0.093				
Natal	30	6197	20	10			0.019	0.016 ± 0.071				
Population	45	54334	33	12			0.062	0.074 ± 0.074				
Density Variable:					1.331	0.249			0.12	68.76	3.2	271^a^
Discrete	71	20486	50	21			0.014	0.008 ± 0.055				
Continuous	26	47904	22	4			– 0.100	– 0.107 ± 0.094				
Dispersal Metric:					1.979	0.372			0.12	69.17	3.24	274^a^
Distance	41	19433	28	13			– 0.004	– 0.002 ± 0.062				
Propensity	36	43539	27	9			– 0.019	– 0.021 ± 0.070				
Rate	20	5418	17	3			– 0.038	– 0.068 ± 0.148				
Temporal Scale:					3.218	0.200			0.11	67.23	3.05	261^a^
Inter-annual	34	20033	28	6			0.036	0.048 ± 0.090				
Intra-annual	51	44807	34	17			– 0.103	– 0.130 ± 0.057				
Per trial	12	3550	10	2			0.204	0.232 ± 0.120				
Spatial Scale:					0.755	0.385			0.12	60.95	3.22	276^a^
Between patches	48	55798	38	10			– 0.004	0.005 ± 0.070				
Out of patch	49	12592	34	15			– 0.029	– 0.040 ± 0.064				
Birds												
25	8359		16	9			– 0.039	– 0.051 ± 0.071	0.02	23.36	1.30	30
Sex:					0.35	0.838			0.03	28.99	1.41	30
Males	7	2442	5	2			– 0.103	– 0.128 ± 0.162				
Females	6	1038	4	2			– 0.081	– 0.085 ± 0.127				
Males + Females	12	4879	7	5			0.020	0.010 ± 0.100				
Age:					< 0.001	1			0.03	26.58	1.36	30
Adults	12	5625	8	4			0.012	0.004 ± 0.096				
Juveniles	13	2734	8	5			– 0.086	– 0.102 ± 0.105				
Migratory Behavior:					0.01	0.932			0.01	8.19	1.09	21
Migratory	15	5081	9	6			– 0.183	– 0.208 ± 0.091				
Non-migratory	10	3278	7	3			0.177	0.183 ± 0.064				
Study Design:					< 0.001	1			0.03	26.27	1.36	30
Natural	22	8111	14	8			– 0.061	– 0.076 ± 0.075				
Manipulated	3	248	2	1			0.124	0.130 ± 0.202				
Density Metric:					0.70	0.704			0.01	13.44	1.16	25
Breeding	11	5083	8	3			– 0.107	– 0.118 ± 0.097				
Natal	12	2038	4	5			– 0.014	– 0.031 ± 0.116				
Population	2	1238	1	1			0.184	0.190 ± 0.142				
Density Variable:					0.098	0.755			0.02	25.00	1.33	27
Discrete	20	7424	12	8			0.049	0.047 ± 0.067				
Continuous	5	935	4	1			– 0.392	– 0.443 ± 0.135				
Dispersal Metric:					5.54	0.060			0.02	22.09	1.28	26
Distance	19	6549	14	5			– 0.110	– 0.28 ± 0.085				
Propensity	5	1726	1	4			0.160	0.162 ± 0.046				
Rate	1	84	1	0			0.320	0.332				
Temporal Scale:					< 0.001	1			0.02	25.38	1.34	30
Inter-annual	18	7976	12	6			0.013	0.009 ± 0.070				
Intra-annual	7	383	4	3			– 0.173	0.207 ± 0.174				
Per trial	0	0	0	0			–	–				
Spatial Scale:					0.31	0.580			0.03	28.25	1.39	30
Between patches	17	6902	12	5			0.045	0.042 ± 0.074				
Out of patch	8	1457	4	4			– 0.218	– 0.250 ± 0.136				
Fishes												
8	31150		6	2			0.043	0.042 ± 0.050	0.02	40.99	1.69	11
Sex:					2.67	0.264			0.01	23.98	1.32	5
Males	2	8738	2	0			0.021	0.021 ± 0.048				
Females	2	20106	2	0			– 0.068	– 0.071 ± 0.204				
Males + Females	4	2306	2	2			0.108	0.109 ± 0.023				
Age:					< 0.001	1			0.01	18.23	1.22	4
Adults	2	26342	2	0			– 0.148	– 0.151 ± 0.124				
Juveniles	6	4808	4	2			0.106	0.106 ± 0.017				
Migratory Behavior:					0.67	0.414			0.01	34.11	1.52	8
Migratory	4	2306	2	2			0.108	0.109 ± 0.023				
Non-migratory	4	28844	4	0			– 0.024	– 0.025 ± 0.090				
Study Design:					–	–			–	–	–	–
Natural	0	0	0	0			–	–				
Manipulated	8	311150	6	2			0.043	0.042 ± 0.040				
Density Metric:					< 0.001	1			0.01	39.18	1.64	9
Breeding	0	0	0	0			–	–				
Natal	2	1474	2	0			0.137	0.138 ± 0.028				
Population	6	29675	4	2			0.011	0.010 ± 0.061				
Density Variable:					0.667	0.414			0.01	34.11	1.52	8
Discrete	4	2306	2	2			− 0.108	− 0.109 ± 0.023				
Continuous	4	28844	4	0			− 0.024	− 0.025 ± 0.090				
Dispersal Metric:					5.333	0.0695			0.02	45.40	1.83	9
Distance	1	286	0	1			0.109	0.109				
Propensity	5	29135	5	0			0.014	0.013 ± 0.079				
Rate	2	1729	1	1			0.081	0.081 ± 0.030				
Temporal Scale:					< 0.001	1			0.02	43.04	1.76	10
Inter-annual	1	1183	1	0			0.110	0.110				
Intra-annual	7	29967	5	2			0.033	0.032 ± 0.056				
Per trial	0	0	0	0			–	–				
Spatial Scale:					0.667	0.414			0.01	34.11	1.52	8
Between patches	4	28844	4	0			− 0.024	− 0.025 ± 0.090				
Out of patch	4	2306	2	2			0.108	0.109 ± 0.234				
Herpetofauna												
10	2618		8	2			− 0.260	− 0.326 ± 0.170	0.20	73.24	3.74	34^a^
Sex:					3.750	0.153			0.07	45.11	1.82	13
Males	3	504	3	0			− 0.554	− 0.706 ± 0.276				
Females	3	485	3	0			− 0.489	− 0.578 ± 0.212				
Males + Females	4	1629	2	2			0.132	0.148 ± 0.173				
Age:					0.039	0.843			0.20	73.66	3.80	31^b^
Adults	8	2425	7	1			− 0.321	− 0.403 ± 0.203				
Juveniles	2	193	1	1			− 0.016	− 0.016 ± 0.184				
Migratory Behavior:					0.625	0.429			0.09	53.89	2.17	17
Migratory	5	849	5	0			− 0.517	− 0.660 ± 0.223				
Non-migratory	5	1769	3	2			− 0.003	0.009 ± 0.157				
Study Design:					< 0.001	1			0.01	62.96	2.70	24
Natural	7	2354	6	1			− 0.446	− 0.549 ± 0.163				
Manipulated	3	264	2	1			0.173	0.195 ± 0.236				
Density Metric:					3.750	0.153			0.07	46.16	1.86	13
Breeding	6	989	6	0			− 0.522	− 0.642 ± 0.158				
Natal	2	193	1	1			− 0.016	− 0.016 ± 0.184				
Population	2	1436	1	1			0.279	0.313				
Density Variable:					0.625	0.429			0.22	71.30	3.48	28^a^
Discrete	9	1253	8	1			0.009	0.009				
Continuous	1	1365	0	1			− 0.290	− 0.363 ± 0.186				
Dispersal Metric:					3.213	0.070			0.02	65.10	2.87	12^a^
Distance	1	1365	0	1			0.009	0.009				
Propensity	4	474	3	1			− 0.102	− 0.104 ± 0.090				
Rate	5	779	5	0			− 0.440	− 0.571 ± 0.309				
Temporal Scale:					1.071	0.585			0.15	68.04	3.13	23^a^
Inter-annual	2	240	2	0			− 0.606	− 0.706 ± 0.076				
Intra-annual	7	2307	5	2			− 0.277	− 0.352 ± 0.184				
Per trial	1	71	1	0			0.549	0.617				
Spatial Scale:					< 0.001	1			0.14	64.24	2.8	21^a^
Between patches	6	831	5	1			− 0.400	0.509 ± 0.259				
Out of patch	4	1787	3	1			− 0.051	− 0.052 ± 0.087				
Invertebrates												
28	10815		22	6			0.141	0.166 ± 0.104	0.23	78.9	5.74	130^a^
Sex:					3.893	0.143			0.24	79.36	4.82	122^a^
Males	4	494	2	2			0.384	0.478 ± 0.248				
Females	7	1522	7	0			0.144	0.185 ± 0.211				
Males + Females	17	8799	13	4			0.082	0.085 ± 0.137				
Age:					< 0.001	1			0.23	78.59	4.67	124^a^
Adults	27	10635	21	6			0.163	0.191 ± 0.105				
Juveniles	1	180	1	0			− 0.470	− 0.510				
Migratory Behavior:					0.318	0.573			0.21	76.34	4.23	108^a^
Migratory	9	5489	6	3			− 0.083	− 0.105 ± 0.130				
Non-migratory	19	5326	16	3			0.246	0.295 ± 0.133				
Study Design:					0.520	0.470			0.24	79.48	4.87	128^a^
Natural	11	5442	8	3			0.073	0.109 ± 0.210				
Manipulated	17	5373	14	4			0.184	0.203 ± 0.111				
Density Metric:					1.768	0.413			0.22	78.04	4.55	116^a^
Breeding	5	1787	5	0			− 0.133	− 0.164 ± 0.231				
Natal	3	320	2	1			0.198	0.202 ± 0.056				
Population	20	8708	15	5			0.200	0.243 ± 0.131				
Density Variable:					0.048	0.827			0.24	79.02	4.77	124^a^
Discrete	20	5712	15	5			0.186	0.215 ± 0.108				
Continuous	8	5103	7	1			0.026	0.044 ± 0.254				
Dispersal Metric:					0.28	0.868			0.20	75.25	4.04	95^a^
Distance	6	2424	2	1			0.244	0.276 ± 0.141				
Propensity	16	6757	12	4			− 0.022	− 0.023 ± 0.150				
Rate	6	1634	5	1			0.470	0.561 ± 0.148				
Temporal Scale:					1.075	0.584			0.10	60.00	2.50	60^a^
Inter-annual	3	1899	3	0			0.772	1.03 ± 0.042				
Intra-annual	15	5788	11	4			− 0.035	− 0.057 ± 0.132				
Per trial	5	3128	8	2			0.215	0.241 ± 0.130				
Spatial Scale:					0.179	0.673			0.23	78.18	4.58	121^a^
Between patches	9	5936	8	1			0.238	0.293 ± 0.239				
Out of patch	19	4879	14	5			0.094	0.106 ± 0.107				
Mammals												
26	15448		20	6			− 0.089	− 0.101 ± 0.086	0.03	34.90	1.54	42^a^
Sex:					1.942	0.379			0.05	44.95	1.82	42^a^
Males	4	2636	2	2			0.049	0.060 ± 0.173				
Females	5	2113	4	1			− 0.308	− 0.369 ± 0.198				
Males + Females	17	10699	14	3			− 0.057	− 0.060 ± 0.109				
Age:					2.612	0.11			0.37	39.53	1.65	42^a^
Adults	14	14097	13	1			− 0.101	− 0.111 ± 0.122				
Juveniles	12	1351	7	5			− 0.074	− 0.090 ± 0.127				
Migratory behavior					1.370	0.242			0.04	38.16	1.62	41^a^
Migratory	7	9108	7	0			− 0.010	0.013 ± 0.145				
Non-migratory	19	6340	13	6			− 0.118	− 0.143 ± 0.106				
Study Design:					< 0.001	1			0.04	40.28	1.67	42^a^
Natural	22	14206	17	5			− 0.089	− 0.103 ± 0.101				
Manipulated	4	1242	3	1			− 0.087	− 0.090 ± 0.106				
Density Metric:					< 0.001	1						
Breeding	0	0	0	0			–	–	0.03	33.61	1.51	40^a^
Natal	11	2172	8	3			− 0.008	− 0.001 ± 0.148				
Population	15	13736	12	3			− 0.148	− 0.175 ± 0.103				
Density Variable:					0.122	0.727			0.04	40.36	1.68	42^a^
Discrete	18	3791	13	5			− 0.086	− 0.100 ± 0.114				
Continuous	8	11657	7	1			− 0.095	− 0.103 ± 0.125				
Dispersal Metric:					3.198	0.202			0.04	38.39	1.62	39^a^
Distance	14	8809	9	5			0.026	0.042 ± 0.116				
Propensity	6	5447	6	0			− 0.136	− 0.142 ± 0.098				
Rate	6	1192	5	1			− 0.309	− 0.394 ± 0.208				
Temporal Scale:					5.720	0.057			0.04	42.10	1.73	41^a^
Inter-annual	10	8735	10	0			− 0.024	0.031 ± 0.198				
Intra-annual	15	6362	9	6			− 0.122	− 0.138 ± 0.077				
Per trial	1	351	1	0			− 0.242	− 0.247				
Spatial Scale:					< 0.001	1			0.03	35.13	1.54	41^a^
Between patches	12	13285	9	3			− 0.049	− 0.037 ± 0.090				
Out of patch	14	2163	11	3			− 0.123	− 0.156 ± 0.142				

**P* significant at$\alpha$= 0.05; ***P* significant at$\alpha$= 0.01

^a^*Q* significant at$$\alpha$$= 0.05
