# Supplementary material for: Study methodology impacts density-dependent dispersal observations: a systematic review
Source: Mov Ecol. 2024 May 21;12:39. doi: 10.1186/s40462-024-00478-6 (PMC11107046; doi:10.1186/s40462-024-00478-6)
Supplement: Supplementary file 1 — Additional file 1. [file 40462_2024_478_MOESM1_ESM.docx]

**
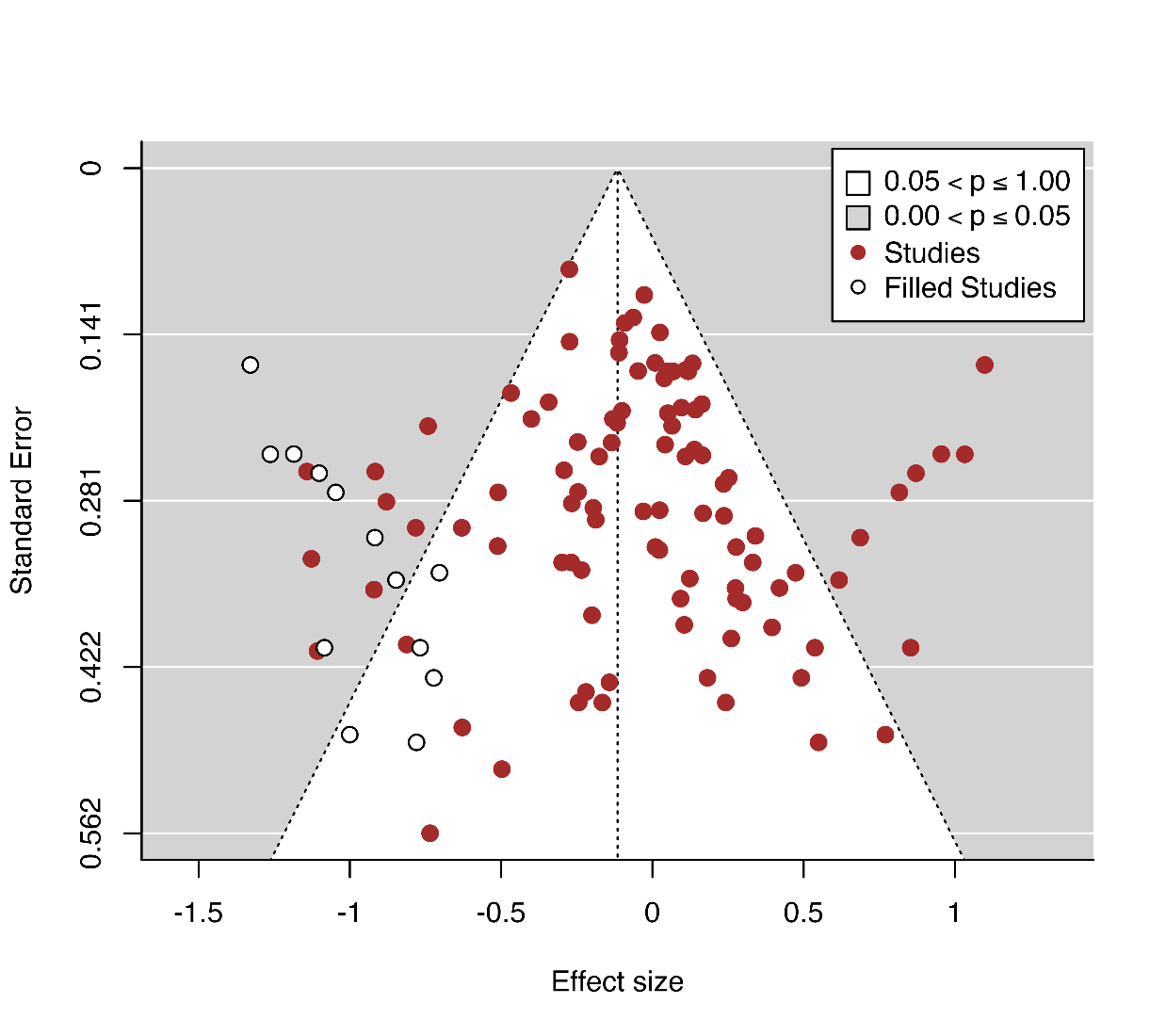
**

**Supplementary Figure S1:** Asymmetry in effect sizes across studies on density-dependent dispersal (*k* = 97) shown in a funnel plot (with trim-and-fill estimation of missing data) of effect size ($Z_{r}$) against standard error, with trim-and-fill estimation of missing data points (open circles).
